# New insights into the cultivability of human milk bacteria from ingestion to digestion and implications for their Immunomodulatory properties

**DOI:** 10.1038/s41598-025-95668-6

**Published:** 2025-03-31

**Authors:** Charles Le Bras, Alizé Mouchard, Lucie Rault, Marie-Françoise Cochet, Olivia Ménard, Nolwenn Jacquet, Victoria Chuat, Florence Valence, Yves Le Loir, Amandine Bellanger, Amélie Deglaire, Isabelle Le Huërou-Luron, Sergine Even

**Affiliations:** 1 STLO, INRAE, Institut Agro Rennes Angers, Rennes, France; 2 Institut NuMeCan, INRAE, INSERM, Université de Rennes, Saint Gilles, France; 3 Pediatric Department, CHU Rennes, CIC-Inserm 1414, Rennes, France

**Keywords:** Breast milk microbiota, Gastrointestinal digestion, Bacterial cultivability, Bacterial viability, Immunomodulatory properties, In vitro digestion, Immunology, Microbiology

## Abstract

Human milk (HM) microbiota is increasingly studied for its potential health benefits. However, the physiological state of HM bacteria and consequently their effects on gut homeostasis remain a question. This study investigated the physiological state of the HM microbiota by characterizing its cultivable fraction as it might be at the point of ingestion and assessing the effects of digestion, in the specific context of the immature infant digestive tract, on the cultivability and immunomodulatory properties of six HM strains representative of prevalent genera in HM. Twenty-eight HM samples were analysed by 16 S metabarcoding either directly on raw milk (raw milk microbiota, RM) or on the complete cultivable fraction obtained from seven non-selective media (cultivable milk microbiota, CM). This approach enabled a more in-depth investigation of CM than conventional methods based on the individual sequencing of a subset of isolates and resulted in a moderate gain in diversity within each HM sample. It confirmed that diversity was lower in CM than in RM, with ~ 7 versus 69 genera per sample in CM and RM respectively, and an under-representation of strictly anaerobic genera in CM. In vitro infant gastrointestinal digestion resulted in overall good survival of the 6 HM strains but partial or complete loss of their immunomodulatory properties on the monocyte THP1 cell line, except for a *Staphylococcus epidermidis* strain that gained immunomodulatory potential. These results highlight the potential of HM bacteria to survive during the infant gastrointestinal digestion and interact with the intestinal epithelium and immune system, as well as the importance of considering the digestion process when evaluating host-bacteria interactions.

**Supplementary Information:**

The online version contains supplementary material available at 10.1038/s41598-025-95668-6.

## Introduction

Human milk (HM) microbiota has been widely explored in recent years, mainly through metagenomic approaches, revealing an initially unsuspected diversity^1–6^. Hence, HM was reported to host a low bacterial load but complex microbial community, with hundreds of bacterial genera and species identified, mainly within the Bacillota, Actinomycetota and Pseudomonadota^2,7^. The most prevalent bacterial species include *Staphylococcus* and *Streptococcus*, that are universally predominant in HM as well as *Cutibacterium*,* Corynebacterium*,* Bifidobacterium*,* Lactobacillus*,* Pseudomonas*,* Rothia*,* Acinetobacter*,* Enterococcus* and several genera known to be strictly anaerobic such as *Faecalibacterium*,* Bacteroides* or *Veillonella*^2–4,7^. A few studies have also investigated this microbial community using culture-dependent approaches, revealing a less diverse bacterial community than with culture-independent approaches, and a limited number of strictly anaerobic bacteria^1,8–11^. These culture-dependent approaches are generally based on the selection of a subset of isolates that are individually identified by sequencing the 16 S rRNA gene or by mass spectrometry analysis^1,11^.

Discrepancies between culture-dependent and independent methods question our ability to capture the microbial diversity of HM with culture-dependent methods, but they also question the physiological state of HM bacteria (either viable in a cultivable or “viable but non-cultivable” (VBNC) state, or dead) and consequently their ability to colonize the infant gut and influence the infant gut microbiota and homeostasis through direct interactions and/or their metabolic activities^12,13^. Nevertheless, several studies support a role of HM microbiota on infant gut homeostasis. Some studies pointed to an overlap between the microbiota of HM and that of the infant gut or, more broadly, to the influence of the first one on the composition of the second one^14–16^. In addition, some strains are shared between HM and the infant microbiota within a dyad, notably strictly anaerobic bacteria^17,18^. HM microbiota is thought to play a wider role in intestinal homeostasis, immune and barrier functions^19^. In vitro characterization of *Bifidobacterium*, *Lactobacillus* and *Streptococcus* species present in HM has highlighted their ability to modulate the immune and barrier functions of epithelial cells^20–23^. Our previous work on a large number of HM strains has demonstrated the functional diversity of these bacteria in a quadricellular model of intestinal epithelium, both in terms of their ability to stimulate the model’s pro- and anti-inflammatory responses, and their capacity to reinforce or diminish the intestinal epithelial barrier^24^.

The question of the physiological state of HM bacteria goes beyond the state at the time of ingestion, as this state can be modified during digestion. In vitro characterization of HM strains is generally carried out using live bacteria derived from fresh cultures, which in all cases have not undergone digestion steps^24^. The ability of bacterial strains to survive in the gastrointestinal tract has been mainly investigated for probiotic strains, especially *Bifidobacterium* and *Lactobacillus* strains^25^. However, strong inter-strain variability has been reported with important loss of cultivability, up to several log^26–28^. Survival to the digestion process remains rather unexplored in the context of HM, which hosts a large bacterial diversity, beyond lactic acid bacteria and bifidobacteria. Furthermore, the digestive tract of newborns is immature and the digestion process differs from adults, with less harsh conditions in infants^29^. Whether these milder digestive conditions in infants may allow a better survival of a large set of HM bacteria with consequences on their ability to interact with the intestinal epithelium remains to be evaluated.

In this study, we hypothesize that HM bacteria could be present in different physiological states (viable or dead) at the time of ingestion and that the infant’s digestion process could have an impact on the bacterial viability and properties, including their immunomodulatory properties. Here, we thus aimed to evaluate the physiological state of the HM microbiota by studying its cultivable fraction at the time of milk expression (i.e. corresponding to the time of ingestion by the infant). Cultivability has been used here as a proxy to estimate the viable fraction of the HM microbiota, albeit imperfectly, as it excludes the so-called VBNC fraction. The total cultivable fraction of 28 HM was recovered from seven non-selective media and sequenced by 16 S rRNA metabarcoding. This complete cultivable HM microbiota was compared to the raw milk microbiota obtained by 16 S rRNA metabarcoding directly on the HM samples. This strategy allowed a more in-depth exploration of the cultivable HM microbiota compared to conventional approaches that rely on the identification of a limited number of isolates per HM sample. It also allowed a more reliable comparison between raw and cultivable milk microbiota because the same identification method was used. In addition to considering cultivability at ingestion, we evaluated the effect of the gastrointestinal digestion process, using an infant in vitro static digestion model, on the cultivability and immunomodulatory properties on the monocyte THP1 cell line, of six different HM strains representative of the prevalent genera in HM^1,11,13,24^. It is essential to mimic the digestive conditions of infants rather than adults in order to accurately study the fate of HM bacteria and their properties in the infant digestive tract. This study provides new insight into this crucial question regarding the physiological state of HM bacteria throughout the digestive tract, confirming, despite a more in-depth investigation, a limited cultivability of the HM microbiota at the time of ingestion and revealing overall good survival of representative strains of HM bacteria during the infant digestion process while their immunomodulatory properties were affected in a strain-dependent manner.

## Results

### A lower diversity in the cultivable milk microbiota as compared to the raw milk microbiota

The first main objective of this study was to assess the complete cultivable milk microbiota (CM) of 28 HM samples (see Supplementary Table S1 for individual sample description and associated metadata, and Supplementary Table S2 for an overview of cohort characteristics) and compare CM with the raw milk microbiota (RM) of the 28 HM samples. For CM, 100 µL of each HM sample was plated on seven non-selective media under aerobic and/or anaerobic conditions in order to promote the growth of the greatest diversity of bacteria. This included a broad-spectrum rich medium, three media known to promote the growth of strictly anaerobic bacteria and two media that promote the growth of fastidious microorganisms and lactic acid bacteria. Of note, incubation was performed at 37 °C for one to three days, which may limit the recovery of microorganisms with different optimal growth temperatures and/or low growth rates. The median population obtained on the different media was 1.45 × 10^3^, 2.67 × 10^3^, 2.84 × 10^3^, 2.16 × 10^3^, 3.35 × 10^3^, 8.1 × 10^2^, 7.40 × 10^2^ cfu/mL on BHI-YEc under aerobic and anaerobic conditions and on BA, YCFA, PYG, WCc, MRSc respectively. For each HM sample, the cultivable fraction obtained under the seven growth conditions was collected by scraping the plates and pooling them before sequencing using 16 S metabarcoding. Thus, it can be estimated that the median total number of colonies analyzed by metabarcoding was ~ 1700 colonies per HM sample (0.1-fold the sum of the median bacterial population (cfu/mL) in the 7 growth conditions). In a related study, based on the same HM samples and aimed at establishing and characterizing a collection of HM isolates, we individually identified a subset of 1245 isolates from the 28 HM samples plated on the same growth conditions as those used in the present study, plus two additional conditions (MRSc and WCc supplemented with mupirocin in order to inhibit the growth of the dominant Staphylococci and promote the isolation of additional taxa). These isolates (~ 40 colonies per HM sample) were individually identified by full-length 16 S rRNA gene sequencing^24^. Thus, the strategy used in the present study allowed the analysis of ~ 43-fold more colonies than the conventional approach used in the aforementioned related study^24^ and, consequently, a more in-depth assessment of the cultivable HM microbiota.

CM, as determined in the present study using 16 S metabarcoding was then compared with the raw milk microbiota (RM) obtained by direct 16 S metabarcoding on the 28 HM samples. This allowed a more reliable comparison between CM and RM as the same identification method was used for both. Overall, our sequencing effort produced a total of ~ 3.3 million quality filtered sequences corresponding to 27 RM and 28 CM (the available volume of one milk sample was too small to allow RM analysis). The median number of sequences was 53 465 sequences per sample (24772 and 91 741 in RM and CM, respectively), corresponding to 1775 bacterial OTUs detected based on a minimum abundance of > 0.005% in the full dataset. The abundance table for each sample is presented in Supplementary Table S3 online, following aggregation at different taxonomic levels.

**Fig. 1:**
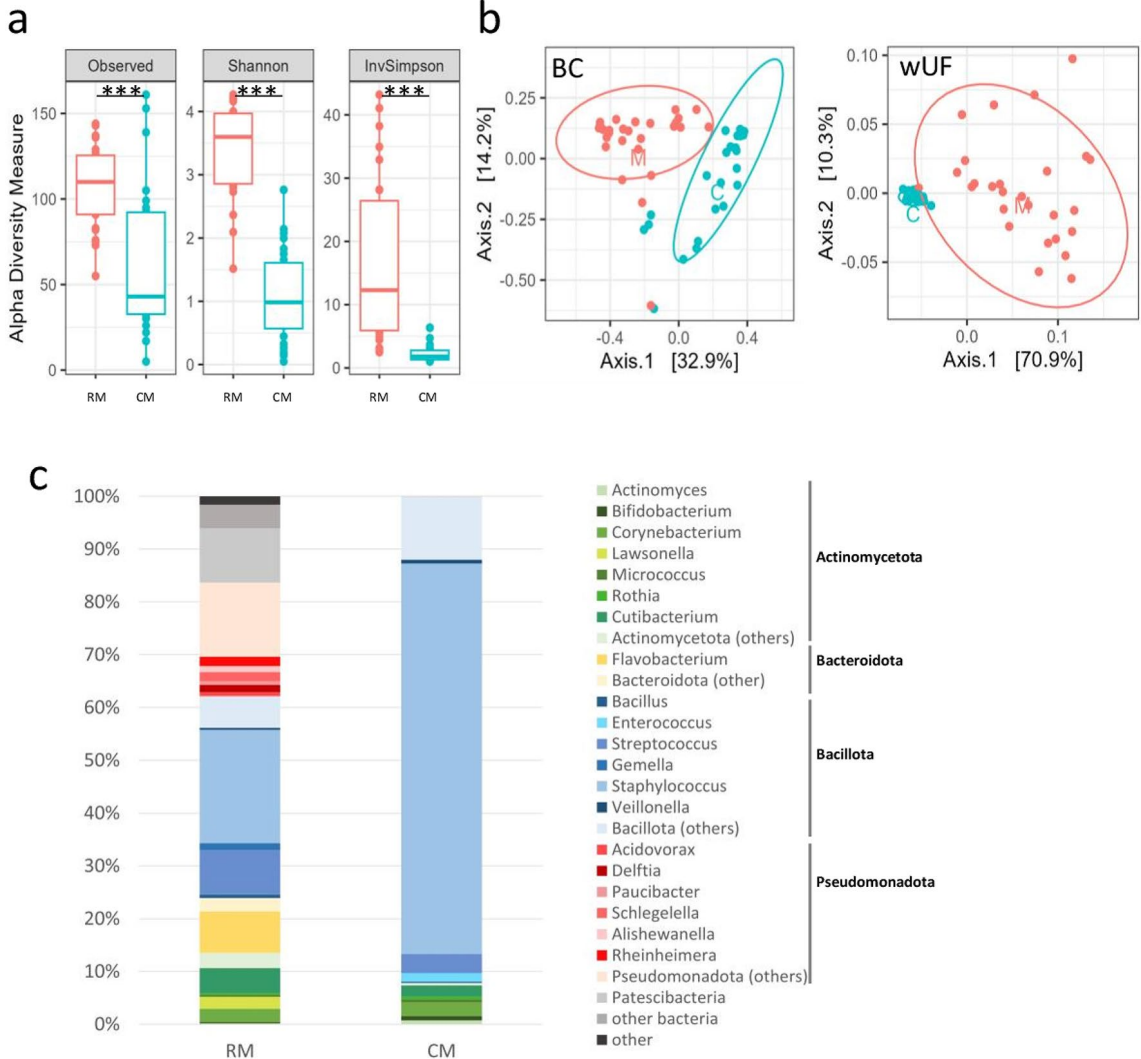
Overview of HM microbiota with regard to the sample type. ( **a**) Alpha-diversity (Observed richness, Shannon and Inverse Simpson index) of raw milk microbiota (RM) (red) and cultivable milk microbiota (CM) (blue). Significant difference in alpha-diversity was observed between RM and CM as determined by ANOVA (***p < 0.001). (**b**) Multi-Dimensional Scaling (MDS) of RM and CM. MDS was performed based on the measurement of the Bray–Curtis (BC) or weighted UniFrac (wUF) distances. Samples are indicated by points and coloured with regard to the sample type: RM (red) and CM (blue). **(c)** Mean taxonomic profiles of RM and CM. The 20 dominant genera are presented as well as the 5 dominant phyla. Genera belonging to Actinomycetota (formerly named Actinobacteria) are displayed in shades of green, Bacteroidota (formerly named Bacteroidetes) in shades of yellow, Bacillota (formerly named Firmicutes) in shades of blue, Pseudomonadota (formerly named Proteobacteria) in shades of red.

A general overview of RM and CM revealed that they strongly differed in terms of both α- and β-diversities (Fig. 1). Hence, the α-diversity of CM was significantly lower than that of RM (*p* < 0.001; Fig. 1a). Similarly, the sample type affected the β-diversity, with a clear separation between RM and CM samples (Fig. 1b). This was confirmed by PERMANOVA analysis (*p* = 1.10^− 4^) with a contribution of sample type to beta-diversity of 17%, 23%, 48% and 53% for Jaccard, Bray-Curtis, Unifrac and weighted Unifrac distances, respectively. The complete RM included 27 phyla corresponding to 435 genera, 605 species and 1231 OTUs whereas 4 phyla, 32 genera, 68 species and 612 OTUs were found in CM (Table 1). Likewise, the median number of genera and OTUs per sample was ~ 10-fold and 2-fold lower in CM compared to RM (69 genera and 96 OTUs/samples for RM and 7 genera and 53 OTUs/sample for CM).

**Table 1:** Overview of the bacterial taxonomic composition of raw milk microbiota (RM) and cultivable milk microbiota (CM). Total number of taxa for each taxonomic level is given as well as the median number per sample^a^.

	All samples	Raw milk microbiota		Cultivable milk microbiota	
	number	number	Median number/ sample	number	median number/ sample
Phyla	27	27	11	4	2
Class	58	58	17	6	2
Order	155	155	43	14	6
Family	252	250	54	23	7
Genera	441	435	69	32	7
Species	634	605	76	68	13
OTU	1759	1231	96	612	53

^a^ only taxa with a total number of reads *≥* 8 (corresponding to a minimum abundance of 0.005%) in a given sample were considered as present in the sample. Likewise, only taxa that were present at least in one CM sample, one RM sample or one sample (either CM or RM) with a number of reads *≥* 8 were considered in the total number of taxa in CM, RM and “all samples”, respectively. Hence, 1759 of the 1775 detected bacterial OTUs were present in at least one CM and/or RM sample with a minimum of 8 reads.

RM was dominated by 5 phyla, namely Bacillota (mean abundance of 38%), Actinomycetota (14%), Pseudomonadota (21%), Bacteroidota (10%) and Patescibacteria (10%), while CM was almost exclusively composed of the two formers with mean relative abundance of 92% and 8% (Fig. 1c). Bacteroidota and Pseudomonadota were almost absent in CM with the exception of *Prevotella*, *Moraxella*,* Klebsiella* and *Haemophilus*. *Staphylococcus*, *Streptococcus*,* Cutibacterium* and *Corynebacterium* were the most abundant and prevalent genera in CM. Additional prevalent and/or abundant genera present in CM included *Enterococcus*,* Bacillus*,* Veillonella*,* Bifidobacterium*,* Micrococcus*,* Kocuria*,* Actinomyces* and *Rothia*, and, to a lesser extent *Lactobacillus*,* Finegoldia*, and *Granulicatella* (Supplementary Figure S1 and Table S3). While all the genera present in CM were present in RM as well, several genera present in RM were not recovered from CM. This included strictly anaerobic genera such as those belonging to the Clostridia and Negativicutes classes, with the exception of *Finegoldia* and *Veillonella*, which were present in CM of few samples (Supplementary Table S3).

Of note, several metadata were associated with the HM collection, including parity, maternal age and body mass index, and maternal supplementation with iron and/or with vitamins and trace elements either during gestation or lactation (Supplementary Tables S1 and S2). While the sample size of this study was sufficient to address the impact of the sample type and thus to compare RM and CM, as confirmed by a statistical power analysis (see Material and methods), it was too small to evaluate the impact of the other factors.

### Infant gastrointestinal digestion affects bacterial cultivability and immunomodulatory properties in a strain dependant manner

The second main objective was to evaluate the impact of the gastrointestinal digestion on the cultivability and immunomodulatory properties of 6 strains previously isolated from HM: *Bifidobacterium breve* CIRM BIA 2845, *Streptococcus salivarius* CIRM BIA 2846, *Cutibacterium acnes* CIRM BIA 2849, *Staphylococcus epidermidis* CIRM BP 1633, *Lactobacillus gasseri* CIRM BIA 2841 and *Enterococcus faecalis* CIRM BIA 2835 ^24^. These strains were chosen to cover, albeit imperfectly, the taxonomic diversity found in HM (which is dominated by Bacillota and Actinomycetota) and they belong to the prevalent genera of HM microbiota (prevalence > 10% in the present study and prevalent genera as reported in the litterature^2–4^). Strains belonging to *Staphylococcus*,* Streptococcus* and *Cutibacterium* were isolated from > 93% of HM samples and corresponded to the 3 highest abundant genera in CM (Supplemental Figure S1), and the first two genera were reported to be universally predominant in HM^2–4^. *Bifidobacterium*,* Lactobacillus* and *Enterococcus* are also prevalent genera of HM and were isolated from 25%, 11% and 21% of HM samples, respectively. *Bifidobacterium* and *Lactobacillus* were also retained for their role in the infant microbiota, as a dominant genus and a primocolonizer respectively, and *Enterococcus* as a commensal, well-adapted inhabitant of the gut environment^30^. The six strains were tested using an in vitro digestion model adapted to the infant stage, in order to estimate more accurately the fate of these strains in the specific context of the immature infant digestive tract^29^. This infant model is characterized by a reduced level of enzymes and bile salts, as well as a higher gastric pH.

**Fig. 2:**
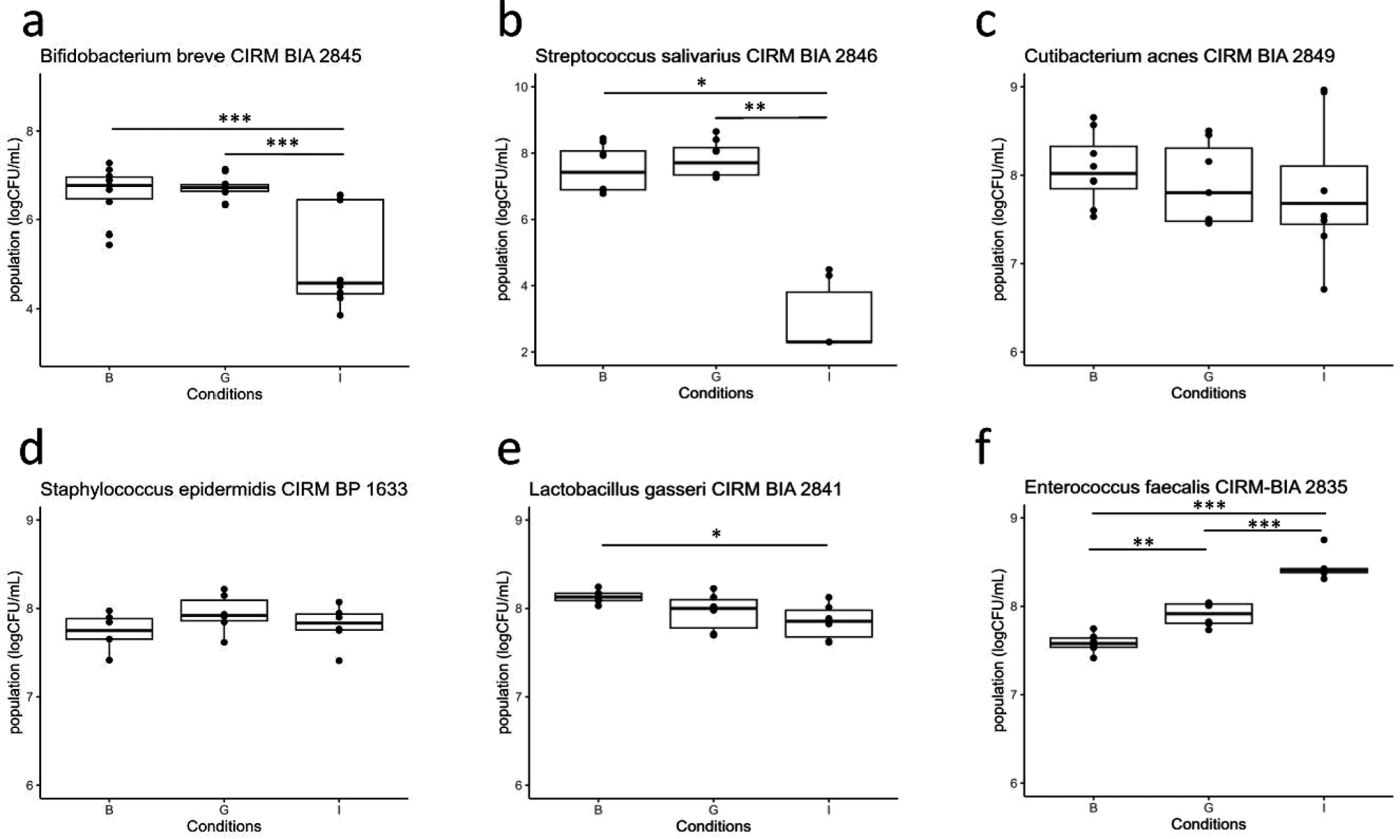
Impact of gastric (G) and gastro-intestinal (I) digestion on the cultivability of six HM strains, as compared to the control before digestion (B). Strains were added to an infant formula and digested under static conditions mimicking the infant gastric (G) or gastric followed by intestinal digestion (I). Cultivable population of each strain was determined on its optimal growth medium. Significant changes in the cultivable population were determined by ANOVA followed by a post-hoc test to find significant pairwise differences. ***P value < 0.001; **P value < 0.01; *P value < 0.05. a; *Bifidobacterium breve* CIRM BIA 2845; b: *Streptococcus salivarius* CIRM BIA 2846; c: *Cutibacterium acnes* CIRM BIA 2849; d: *Staphylococcus epidermidis* CIRM BP 1633; e: *Lactobacillus gasseri* CIRM BIA 2841; f: *Enterococcus faecalis* CIRM BIA 2835.

The gastric phase of digestion had no significant impact on bacterial population, with the exception of *E. faecalis* whose population slightly increased (0.3-log) (Fig. 2). On the contrary, the intestinal phase impacted the bacterial cultivability in a strain-dependant manner, with a decrease of population of 2-log, 4.5-log and 0.3-log for *B. breve*, *S. salivarius* and *L. gasseri* respectively, a 0.7-log increase for *E. faecalis* compared to the initial population and no impact for *C. acnes* and *S. epidermidis* (Fig. 2). All strains retained at least partial cultivability after the gastrointestinal digestion.

**Fig. 3:**
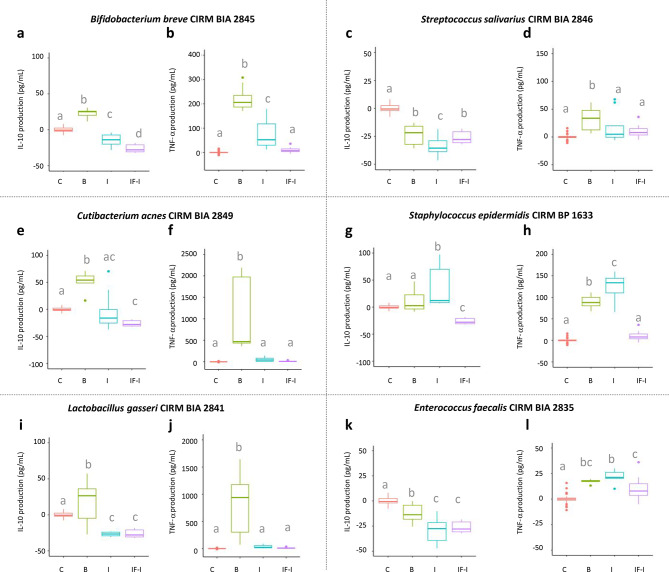
Impact of gastro-intestinal digestion on the immunomodulatory properties of six HM strains. Strains were digested in infant formula under static conditions mimicking the neonate gastric followed by intestinal digestion, recovered by centrifugation before assessing their immunomodulatory properties on THP1 cells through the impact on IL-10 (a, c, e, g, I, k) and TNF-α (b, d, f, h, j, l) secretion. Bacteria, either before (B) and after gastro-intestinal digestion (I), were added at a MOI of 10 bacteria per cell. IL-10 and TNF-α production (pg/mL) were corrected by the production in the absence of bacterial stimulation (C, control) of the corresponding replicate. An additional control corresponded to the digestion of the infant formula without the addition of bacteria (IF-I). Significant changes in IL-10 or TNF-α production were determined by ANOVA followed by a post-hoc test to find significant pairwise differences. Different letters correspond to conditions that were significantly different (P value < 0.05). a, b ; *Bifidobacterium breve* CIRM BIA 2845; c, d: *Streptococcus salivarius* CIRM BIA 2846; e, f: *Cutibacterium acnes* CIRM BIA 2849; g, h: *Staphylococcus epidermidis* CIRM BP 1633; i, j: *Lactobacillus gasseri* CIRM BIA 2841; k, l: *Enterococcus faecalis* CIRM BIA 2835.

We further investigated the effect of digestion on the immunomodulatory properties of the 6 strains on THP1 cells differentiated into macrophages, through the production of IL-10 and TNF-α. We used two different controls, cells without bacterial stimulation (B) and cells stimulated with the digested formula alone without bacteria (IF-I). The digested infant formula had no effect on TNF-α release by THP1 cells, but induced a slight decrease in IL-10 release (Fig. 3). The immunomodulatory properties were modulated by the digestion process in a strain-dependent manner. *C. acnes and L. gasseri* completely lost their immunomodulatory properties, as shown by the absence of differences in IL10 and TNF-α production between cells stimulated with either the digested strain (I) or the digested IF alone (IF-I). (Fig. 3e, f,i, j). After gastrointestinal digestion, *E. faecalis* was still able to slightly stimulate TNF-α secretion but had no more effect on IL-10 production compared to the digested formula alone (Fig. 3k, l). Conversely, S. *salivarius* still very slightly but significantly decreased IL-10 production compared to the digested formula alone but had no more effect on TNF-α secretion (Fig. 3c, d). *B. breve* partially retained its ability to stimulate IL-10 and TNF-α compared to the digested formula alone (Fig. 3a, b). Finally, *S. epidermidis* gained the ability to slightly stimulate IL-10 and increased its stimulation of TNF-α production (Fig. 3g, h).

## Discussion

Although long ignored, the milk microbiota is now considered to be a component of HM that may contribute to infant gut microbiota and homeostasis^12,13^. Despite numerous studies on HM microbiota, the question of the physiological state of HM bacteria that may influence gut colonisation and interactions with the host, remains largely unanswered. In this study, we addressed this issue from ingestion to the end of the small intestine, by exploring the complete cultivable fraction of fresh HM and assessing the effect of gastrointestinal digestion on the cultivability and immunomodulatory properties of 6 prevalent HM microbiota species. The cultivable HM microbiota is a subset of the viable HM microbiota. Some viable microorganisms of the HM may not be recovered under the growth conditions used and constitute the VBNC fraction. Bacterial viability is a complex issue that encompasses a continuum of physiological states related to bacterial cell integrity, the ability of strains to grow (under the optimal laboratory growth conditions or the actual conditions) or to be metabolically active. While the ability to grow under actual gut conditions is a prerequisite for gut colonisation, non-growing and non-living bacterial cells may still interact with the host, to some extent, as reported for heat-killed probiotic strains^31^.

The assessment of the cultivable HM microbiota by 16 S metabarcoding, as performed in the present study, allowed a more in-depth exploration of the cultivable fraction of HM than conventional strategies based on individual identification of a subset of isolates^1,11,24^. Our strategy led to the identification of 32 genera and 68 species in total, with a median number of 7 genera and 13 species per HM sample. In a related study, based on the same HM samples (see Results section for more details), we individually identified a subset of 1245 isolates by full-length 16 S rRNA gene sequencing. These 1245 isolates corresponded to a total of 26 different genera and 59 species, with a median number of 5 genera and 7 species per HM sample^24^. Thus, the complete assessment of CM by metabarcoding as performed in the present study, resulted in a moderate overall gain in diversity compared to the more conventional approach of individually identifying a subset of isolates^24^, especially when considering the bacterial diversity per sample (~ twice the number of species per sample). Of note, a moderate increase (~ 8.5%) in the diversity of fecal microbiota has previously been reported using culturomics when picking and identifying all colonies on the plate compared to “experienced” colony picking (i.e., picking 2–3 colonies per colony type on each plate)^32^. Our results on the diversity of CM are in the same range as the total diversity of CM reported by Treven et al., who combined culture-based methods with MALDI-TOF mass spectrometry identification and recovered 25 genera from 1086 isolates from 31 HM samples^11^. Using MALDI-TOF mass spectrometry, Wang et al. identified 14 genera and 34 species from > 3500 colonies from 9 fresh HM samples^33^.

Despite the more in-depth assessment of the cultivable fraction of HM by metabarcoding as compared to a more conventional culturomic approach, which allowed some gain in diversity in each sample, our results confirm the low bacterial diversity in CM compared to RM. A total of 435 genera were recovered in RM from the 28 HM samples, with a median of 69 genera per sample. This high bacterial richness in HM has been widely documented and the main taxa identified here were in good agreement with the literature^2–5,7^. However, several taxa present in the RM were not recovered from the CM, as illustrated by the ~ 14-fold lower total number of genera and the ~ 10-fold lower median number of genera per sample in CM compared to RM. CM was mainly composed of Bacillota and Actinomycetota, with *Staphylococcus*,* Streptococcus*,* Cutibacterium* and *Corynebacterium* being the most abundant and prevalent genera in CM, in agreement with previous studies^1,11,33,34^. Other prevalent genera in CM included *Bacillus*,* Granulicatella*,* Bifidobacterium*,* Enterococcus*,* Veillonella* as well as *Lactobacillus*,* Kocuria*,* Micrococcus*,* Rothia*,* Finegoldia*, and *Actinomyces*, which have also been reported in the cultivable fraction of milk^7,33^. It should be noted that the prevalence of genera was generally consistent between RM and CM or higher in RM than CM (Supplementary Fig. 1), as previously reported^11^, suggesting that some species or strains within the genera commonly found in RM and CM were not cultivable under our growth conditions.

A major difference between RM and CM was the absence in CM of several strictly anaerobic genera, belonging to the phyla Pseudomonadota and Bacteroidota or to the Classes Clostridia and Negativicutes within Bacillota, although we used several media known to promote the growth of anaerobic bacteria^10^. In addition, precautions were taken to preserve this anaerobic flora, as milk samples collected by the mothers were stored in an anaerobic bag immediately after sampling. Even a brief exposure to oxygen during sample processing or breastfeeding may have been sufficient to kill these highly oxygen-sensitive bacteria. We also cannot rule out the possibility that they were already in a non-living or non-cultivable state in HM. Nevertheless, Schwab et al. reported an improved isolation of obligate anaerobic species, including strictly anaerobic Bacteroidota or Clostridia, after milk storage for 6 days at 4◦C, suggesting that these taxa are part of the cultivable fraction of HM microbiota^10^. Similarly, greater bacterial diversity, particularly within the anaerobes, was reported by Wang et al. after enrichment of 9 fresh HM in blood culture medium for 3–6 days (21 genera and 54 species compared to 14 genera and 34 species identified in fresh milk)^33^. High-throughput culture approaches could help to isolate this oxygen-sensitive flora, based on the culturomic approaches developed for gut microbiota and genomic analysis of the nutrient requirements of HM microbiota^35^.

The second main objective of this study was to evaluate the ability of six strains belonging to prevalent HM genera to survive during the infant digestion process, which was achieved thanks to the use of a static digestion model specifically calibrated for infants^29^. Of note, the strains were digested in an infant formula, to mimic HM to some extent, as the matrix has previously been shown to affect the strain survival during digestion^26,28,36^. Cultivability of the six strains were hardly affected by the gastric phase of digestion but it was affected by the intestinal phase of digestion in a strain-dependant manner, with *S. salivarius* and *B. breve* being most affected, *L. gasseri*, *C. acnes* and *S. epidermidis* being little or not affected, and with a slight increase in *E. faecalis* population. Strain differences in survival to digestion process have been reported previously, mainly for lactic acid bacteria such as *Lactobacillus* and *Bifidobacterium*^26–28,37^. The good survival of lactic acid bacteria in the digestive tract is related to the ability of strains to adapt to acid stress or counteract the effects of bile salts, especially in relation to the expression of bile salt hydrolases^38–40^. Good survival and even growth was expected from *E. faecalis*, known as a commensal inhabitant of the human gut^30^. More interestingly, this study highlighted that non-lactic acid bacteria and non-gut-associated bacteria such as *Cutibacterium acnes* and *Staphylococcus epidermidis* retained high cultivability under the harsh physiological conditions encountered in the stomach and small intestine related to low pH and secretion of bile salts. In line with this, good survival was previously reported for a strain of *Propionibacterium freudenreichii* in different dairy matrices, *Cutibacterium* and *Propionibacterium* being closely related taxonomically^41^. Likewise, Adouard et al. evaluated the cultivability of a mixture of six bacteria and three yeasts from the microflora of surface-ripened cheeses in a three-compartment dynamic gastrointestinal digester (DIDGI). Compared to the other bacteria, a *Staphylococcus equorum* strain, in pure culture, showed an intermediate resistance to the whole digestion process, with a survival rate lower than *Hafnia Alvei*, similar to that of *Corynebacterium casei* and *Brevibacterium aurantiacum*, and higher than *Lactococcus lactis* and *Arthrobacter arilaitensis*^42^. It should be noted that among *Staphylococcus* species, *Staphylococcus aureus* has several acid resistance mechanisms^43^, as well as a bile efflux pomp and a bile-induced oxidoreductase that may mediate cholate resistance and contribute to survival under human colonic conditions^44^.

Although the digestion process affected the cultivability of some strains, all strains were still partially cultivable after gastrointestinal digestion, with populations between 3 and 8.5 Log CFU/mL. The digestive tract of infant is still immature, with a higher gastric pH and a lower bile salt concentration than in adults^29^. Thus, due to the less stressful conditions in the infant digestive tract, most ingested bacteria are likely to retain some viability and, subsequently, have a greater impact on the gut homeostasis than in adults, by colonizing the gut environment and/or interacting with the gut epithelium. It should be noted that the 6 HM strains used here were grown under optimal laboratory growth conditions before being subjected to the digestion process, and the effect of digestion on these strains in freshly expressed HM could be different in relation to their physiological state under these real-life conditions. Another limitation of the static model used here is that it mimics the digestion process through two sequential stages (the gastric and intestinal phases) and does not take into account the dynamics of changes throughout the digestive tract, in terms of pH and digestive enzymes. The use of a dynamic model of digestion such as the DIDGI^45^ would allow a more reliable assessment of the effect of digestion on the cultivability of bacterial strains. Nevertheless, the changes in conditions experienced by the bacteria in this static model are more drastic and therefore likely to be more stressful than in a dynamic model or in vivo where they are more gradual, suggesting that most of the strains used in this study would retain partial cultivability under dynamic digestion.

Despite a limited impact on strain cultivability, a partial or complete loss of immunomodulatory properties was revealed for most strains after digestion, with the exception of *Staphylococcus epidermidis*, which gained the ability to stimulate IL-10 production, and showed increased stimulation of TNF-α production. A loss of its anti-inflammatory properties was also reported for *Propionibacterium freudenreichii* after digestion^41^. This loss is likely due to proteolysis of bacterial cell surface proteins involved in cell stimulation by digestive enzymes such as trypsin. In our study, the loss of immunomodulatory properties was not necessarily associated with the cultivability after gastrointestinal digestion, as illustrated by *C. acnes*, which completely lost its immunomodulatory properties, while cultivability was unaffected (Figs. 2 and 3). Nevertheless, good survival can help to counteract the effects of proteolysis and allow *de novo* biosynthesis of new cell wall proteins. Moreover, bacteria still cultivable after gastrointestinal digestion could theoretically grow or at least be metabolically active in the lower part of the digestive tract, where conditions are “friendlier”. The differences between strains could be related to the nature of the determinants involved in the interaction with THP1 cells such as proteins but also exopolysaccharides, teichoic acids, peptidoglycans or metabolites^46–48^. Regarding *S. epidermidis*, it is possible that its immunomodulatory effects are related to non-protein components, or that the digestion of some surface components (proteins, polysaccharides) has uncovered other cell-interacting components, thus modifying the interplay with some intestinal cell receptors. From a more general point of view, this study highlights the need to take into account the effect of the digestion process not only on the cultivability but also on the properties of bacterial strains, given the poor relationship between these two parameters.

In conclusion, the complete assessment of the HM cultivable microbiota by metabarcoding, as proposed in the present study, enabled a moderate gain in diversity within each HM sample compared to a more conventional approach based on the individual identification of a subset of isolates. Nevertheless, we confirmed that the diversity of the CM was limited compared to that of the RM. Furthermore, the gastrointestinal digestion process, as it occurs in the immature infant digestive tract, affected bacterial cultivability in a strain-dependent manner, with overall good maintenance of cultivability for most HM strains including non-lactic acid bacteria, or non-gut-associated bacteria. This suggests that most of them could grow and/or be metabolically active in the small intestine and later in the colon. Similarly, the immunomodulatory properties of HM bacteria were modulated in a strain-dependent manner, with no direct relationship to cultivability. Most of the strains tested partially or completely lost their immunomodulatory properties on THP1 cells, with the exception of a *Staphylococcus epidermidis* strain, which gained some immunomodulatory capacity. These results highlight the potential of HM bacteria, regardless of their taxonomy, to interact with the intestinal immune system, and the importance of considering the digestion process when addressing the question of their interactions with the intestinal epithelium and mucosal immune system.

## Materials and methods

### Ethics declaration

The HM sampling protocol was carried out in accordance with relevant guidelines and regulations and was approved by the Institutional Review Board of the Poitiers Hospital (n°20.05.27.67526). Informed consent was obtained from all participants.

### HM sampling and preparation of the cultivable HM fraction

HM sampling has been described previously^24^. Mothers were recruited at the University Hospital Center of Rennes during the first week following delivery. HM sampling was performed between 2.0 and 6.0 weeks post-partum as previously described^24^, on twenty-eight healthy mothers who delivered a healthy baby vaginally at term and exclusively breastfed their infant. Exclusion criteria were any formula feeding in addition to breastfeeding, signs of infection or administration of drugs (including antibiotics) to the baby and to the mother during the 3 months prior to delivery and during lactation. Before sampling, the breast was thoroughly washed with water and soap and rinsed with sterile physiological water (0.9% NaCl) before cleaning with DakinR (Sodium Hypochloride 0.5%) and individual sterile compress. Twenty mL of milk were then collected using a new and sterilized manual breast pump (Medela, Issy-les-Moulineaux, France). To minimize the exposure to O2, samples were stored in an anaerobic bag for a maximum of 18 h until use. HM samples were stored at -80 °C for direct analysis of the raw milk microbiota (RM). In addition, each fresh HM sample was used for the determination of the cultivable milk microbiota (CM). HM samples (100 µL) were plated on seven different non-selective media to promote the growth of the greatest diversity of bacteria: BHI-YEc, the non-selective Brain Heart Infusion medium (BD, Franklin Lakes, NJ, USA) supplemented with 1% yeast extract (Biokar, Pantin, France) and 0.05% L-cysteine hydrochloride (Sigma-Aldrich, St. Quentin Fallavier, France) to promote the growth of all HM bacteria; the Columbia II medium containing 5% sheep blood (BD, Le Pont de Claix, France) (BA), which promotes the growth of fastidious microorganisms; the Yeast extract Casitone and Fatty Acids (YCFA), the Peptone Yeast Glucose (PYG) modified medium, and the Wilkins Chalgren (BD, Franklin Lakes, NJ, USA) medium supplemented with 0.05% L-cysteine-hydrochloride (WCc), these three media favouring the growth of anaerobic bacteria; and the Man Rogosa Sharp (BD, Franklin Lakes, NJ, USA) medium supplemented with 0.05% L-cysteine-hydrochloride (MRSc) to favour the growth of Lactobacilli. All media were incubated at 37 °C in anaerobic jars for 1 to 3 days, except BHI-YEc which was incubated in both aerobic and anaerobic conditions. All the colonies on the surface of each medium were collected by scrapping the plate, resuspended in 500 µL of 0.9% NaCl solution, pooled and centrifuged (10,000 g, 5 min, 4 °C) and the pellet was stored at -80 °C until microbiota analysis by metabarcoding in order to determine the complete CM.

### Raw and cultivable milk microbiota analysis

Microbiota analysis by metabarcoding was performed on RM and CM. Raw milk samples (3 mL) were thawed on ice, mixed with 1 mL of sodium citrate (1 M, pH 7.5) and centrifuged (18,000 g, 20 min, 4 °C). After washing with 1 mL of sodium citrate (20 g/L, pH 7.5) and centrifugation (18,000 g, 15 min, 4 °C), the pellet was resuspended in 100 µL TE buffer (10 mM TRIS–HCl (pH8), 2 mM EDTA). For CM, frozen pellets were resuspended directly in 100 µL TE buffer.

DNA extraction was performed as described by Mariadassou et al.^49^, . Briefly, the bacterial suspension was lysed in 400-µL lysis buffer containing 20 mM TRIS–HCl (pH 8), 2 mM EDTA, 1% Triton X100 and 0.4 g of 0.1 mM zirconium beads (VWR, Fontenay-sous-Bois, France) for 3 × 30 s at 6800 rpm by using a Precellys Evolution device (Bertin Technology, Montigny-le-Bretonneux, France). Following incubation at 95 °C for 7 min, samples were mixed for 15 s and centrifuged (18,000 g, 5 min, Room Temperature). Proteinase K treatment and DNA purification were performed using the Qiagen kit QIAamp Fast DNA stool mini kit (Qiagen Courtaboeuf, France), according to the manufacturer’s recommendations.

PCR amplification using the universal primers S-D-Bact-0341-b-S-17 and S-D-Bact-0785-a-A-21 targeting the V3-V4 region of the gene encoding 16 S rRNA and amplicon sequencing on the Illumina MiSeq PE250 platform (Illumina Inc., San Diego, CA, USA) were performed by Genome Quebec (Montreal, Canada) exactly as previously described^49^. Negative controls that underwent all steps from extraction to sequencing but without bacterial suspension were included for each set of extractions, resulting in 6 negative controls.

Sequence library analysis was performed using the FROGS pipeline hosted on the INRAE MIGALE bioinformatics platform essentially as previously described^50^. Briefly, the preprocessing, clustering and chimera removal steps were performed using the FROGS pipeline (Galaxy version 3.2.3 + galaxy2) as previously described^49^. The FROGS clustering step was performed using Swarm with an aggregation distance of 1, allowing for one mismatch between clustered sequences^51^. The data were filtered using the FROGS OTU filter tool, to retain OTUs with a minimum proportion of 0.00005 in the complete dataset. Affiliation was then performed with the FROGS affiliation OTU tool based on Blastn + using the 16 S SILVA 138.1 database^52,53^. The median number of sequences was 53 465 sequences per sample. Negative controls reached a median number of 25 sequences per sample, which was considered negligible compared to milk samples. Negative controls were therefore not included in the analysis. Finally, a rooted phylogenetic tree was created with FastTree and Phangorn R package implemented on FROGS pipeline.

Additional filters were considered to determine the number of taxa (from phylum to OTU) in each sample and prevent its overestimation. Only taxa with a total number of reads *≥* 8 in a given sample were included in the number of taxa in that sample (this corresponded to a minimum proportion of 0.00005 in the sample with the highest number of reads). Likewise, only taxa that were present at least in one CM or one RM sample with a number of reads *≥* 8 were considered in the total number of taxa in CM and RM, respectively.

### Digestion of selected strains in an infant formula matrix

Six strains isolated from the same HM samples were used, namely *Bifidobacterium breve* CIRM BIA 2845, *Streptococcus salivarius* CIRM BIA 2846, *Cutibacterium acnes* CIRM BIA 2849, *Staphylococcus epidermidis* CIRM BP 1633, *Lactobacillus gasseri* CIRM BIA 2841 and *Enterococcus faecalis* CIRM BIA 2835, (hereafter referred to without the strain number)^24^.

Strains were cultivated at 37 °C under static condition for 1 to 3 days, in MRSc for *B. breve* and *L. gasseri* in anaerobic jar, in PYG for *C. acnes* in anaerobic jar, in Tryptic soy broth (Biokar, Pantin, France) supplemented with 1% yeast extract (TSE) for *E. faecalis* and *S. salivarius*, and in BHI-YEc for *S. epidermidis.* The bacterial concentration of the culture was then determined by measuring the optical density at 600 nm (VWR V-1200 Visible Spectrophotometer, VWR, Rosny-sous-Bois, France) and using a previously established OD/CFU relationship. Bacteria were centrifuged (6800 g, 5 min, RT), washed in HBSS 1X (Dutscher, Bernolsheim, France), centrifuged (6800 g, 5 min, RT) and resuspended in RPMI 1640 containing glutamine but without antibiotics or SVF, at a concentration of 1 × 10^9^ CFU/mL. Part of this suspension was used to assess the effect of the strain on THP1 cells before digestion (condition “B”).

The remaining suspension was centrifuged (6800 g, 5 min, RT) and resuspended in an infant formula (IF) (composition for 100 mL as follows: 3.43 g proteins, 8.42 g lipids, 6.21 g carbohydrates, 1.34 g ashes and vitamins) at a bacterial concentration of 3.8 × 10^7^ CFU/mL before digestion using an in vitro model in static conditions at the full-term infant stage^29^. This method mimics in vitro the enzymatic and physicochemical parameters of gastro-intestinal digestion of breast milk in a full-term infant (28 days) weighing approximately 4.25 kg. Two phases were simulated. For the gastric phase, IFs containing bacteria were digested for 1 h at pH 5.3, with simulated gastric fluid (94 mM sodium chloride and 13 mM potassium chloride), considering a ratio (v/v) of meal to secretions of 63 to 37, and porcine pepsin (268 U/mL, Sigma Aldrich). Lipase was not used in this model due to limited commercial availability. After 1 h of gastric digestion, the pH was increased to 7 by addition of NaOH 1 M in order to stop gastric enzyme activities. These digesta were then digested for 1 h at pH 6.6 in a simulated intestinal fluid (164 mM sodium chloride, 10 mM potassium chloride, 85 mM sodium bicarbonate, 3 mM Calcium chloride), considering a ratio (v/v) of meal to secretions of 39 to 61; containing bovine bile (3.1 mmol/L, Sigma Aldrich) and porcine pancreatin (lyophilized pancreatic juice containing trypsin, chymotrypsin and lipase (90 U/mL), Sigma Aldrich) to mimic the intestinal digestion^29^. Digestion was performed in three independent biological replicates, each with two technical replicates, as recommended^29^.

Bacterial cultivability was determined after the gastric and intestinal digestions (conditions “G” and “I” respectively) directly on the digesta using the media described above and the micromethod as previously described^54^.

The remaining digesta was then centrifuged (6800 g, 10 min, RT), washed once with HBSS (6800 g, 10 min, RT) and resuspended in RPMI 1640 containing glutamine but without antibiotics or SVF. The theoretical concentration of this bacterial suspension was 1 × 10^9^ CFU/mL, with no effect of digestion on the cultivability of the strain. A control corresponding to the bacteria-free IF was also digested, washed (under the same conditions as the bacterial pellet) and used for interaction with THP1 cells to assess the immunomodulatory effect of the digested matrix (condition “IF-I”; see below).

### Immunomodulatory properties of bacteria on THP1 cells differentiated into macrophages

The immunomodulatory potential of bacteria was assessed on the monocyte THP1 cell line (ATCC TIB-202). Cells were grown in RPMI1640 (Merck Sigma, Darmstadt, Germany) containing glutamine, supplemented with 10% SVF (decomplemented by heating for 30 min at 56 °C) (Biowest, Riverside, United States), 100 IU/mL penicillin, and 100 µg/mL streptomycin (Merck Sigma) (hereafter referred to as RPMIc). Cells were grown at 37 °C in a 5% CO2 water-saturated atmosphere in 25–75 cm^2^ flasks (Corning Inc, Corning, NY, USA) and diluted to 3 × 10^5^ cells/mL in RPMIc every 3 days. THP1 cells were differentiated into macrophages two days before use. Briefly, 5 × 10^5^ cells/well were seeded in 48-well plates and incubated in RPMIc with 200nM PMA (Phorbol 12-myristate 13-acetate, Merck Sigma, Saint Quentin Fallavier, France). After 24 h, cells differentiated into macrophages were adherent. They were washed 3 times with HBSS (Merck Sigma, Saint Quentin Fallavier, France) before adding 500 µl RPMIc for a further 24 h incubation before the interaction with bacteria.

The bacterial suspension at 1 × 10^9^ CFU/mL, either before digestion or after recovery from intestinal digestion, was diluted at 2 × 10^7^ CFU/mL in RPMIc. THP1 cells (5 × 10^5^ cells/well) were stimulated at a multiplicity of infection (MOI) of 10:1 bacteria per cell, for 24 h in RPMIc at 37 °C in a 5% CO_2_ water saturated atmosphere. The supernatant was centrifuged (8000 g, 5 min, 4 °C) and, after addition of anti-protease cocktail 1X (SigmaFast, Merck Sigma, Saint Quentin Fallavier, France), stored at -20 °C until cytokine analysis by ELISA assay. Experiments were performed in three independent biological replicates, each with two technical replicates, as recommended^55^.

IL-10 and TNF-α were then determined by ELISA (Human IL-10 ELISA Set, Cat. No. 555157; Human TNF-α ELISA Set, Cat. No. 555212; TMB Substrate Reagent Set, Cat. No. 555214) according to the manufacturer’s recommendations. Washes were performed using a biotek 50TS8V 8-channel microplate washer (Agilent Technologies, Les Ulis, France), and absorbance at 450 nm was measured by the biotek 800TS microplate reader (Agilent Technologies, Les Ulis, France). IL-10 and TNF-α production (pg/mL) were corrected by the production in the absence of bacterial stimulation (C, control) of the corresponding replicate.

### Statistical analysis

Statistical analyses on microbiota were performed using R 4.3.0 and specialized packages: phyloseq (v. 1.34), DESeq2 (v 1.30.1) and custom scripts^49,56–58^. The analysis on α-and β-diversity indices was performed on data rarefied to the same depth. Statistical analyses were performed considering the sample type factor, either raw milk (RM) or cultivable milk (CM) sample. Several additional factors were recorded on milk donors, including parity, mother’s age height, weight and body mass index and maternal supplementation with iron and/or with vitamins and trace elements either during gestation or lactation (Supplementary Table S1). A statistical power analysis was performed a posteriori on our dataset using the R pwr and mpress packages^59^ to determine the minimum number of samples required to address the impact of all these factors on RM and/or CM microbiota α-and β-diversities respectively. The sample size of this study was confirmed to be sufficient to investigate the impact of the main factor, sample type, on α- and β-diversities (*n* = 14 and *n* = 5 samples required in each group (RM and CM) for α- and β- diversities respectively). However, the number of samples used in this study was too small to assess the impact of other factors.

Observed, Shannon and Inversed Simpson indices were used to represent the α -diversity. The impact of sample type on each index was assessed using an analysis of variance (ANOVA) (*p* < 0.05). Beta diversity analyses were performed using the UniFrac, weighted UniFrac, Bray-Curtis and Jaccard distances. MultiDimensional Scaling (MDS) was performed on the weighted UniFrac and Bray-Curtis distance matrix to represent the samples on the principal plane. The effect of the different factors on β-diversity was assessed using multivariate analysis of variance (PERMANOVA), as implemented in the adonis2 function from the vegan package.

Regarding the effect of digestion on bacterial cultivability and immunomodulatory properties, statistical analysis was performed using R software (version 4.3.0 ^58^). Differences between groups were examined using a one-way analysis of variance (ANOVA), followed by the Tukey significant difference test to compare group means. Differences were considered statistically significant at *p* < 0.05.

## Electronic supplementary material

Below is the link to the electronic supplementary material.

Supplementary Material 1

Supplementary Material 2

Supplementary Material 3

Supplementary Material 4

## Data Availability

Data is provided within the manuscript or supplementary information files, except data files related to metagenomic analysis that are available at the Sequence Read Archive of the National Center for Biotechnology Information under the accession number PRJNA1184131.
