# Supplementary material for: New insights into the cultivability of human milk bacteria from ingestion to digestion and implications for their Immunomodulatory properties
Source: Sci Rep. 2025 Mar 31;15:10985. doi: 10.1038/s41598-025-95668-6 (PMC11958788; doi:10.1038/s41598-025-95668-6)
Supplement: Supplementary file 1 — Supplementary Material 1 [file 41598_2025_95668_MOESM1_ESM.pdf]

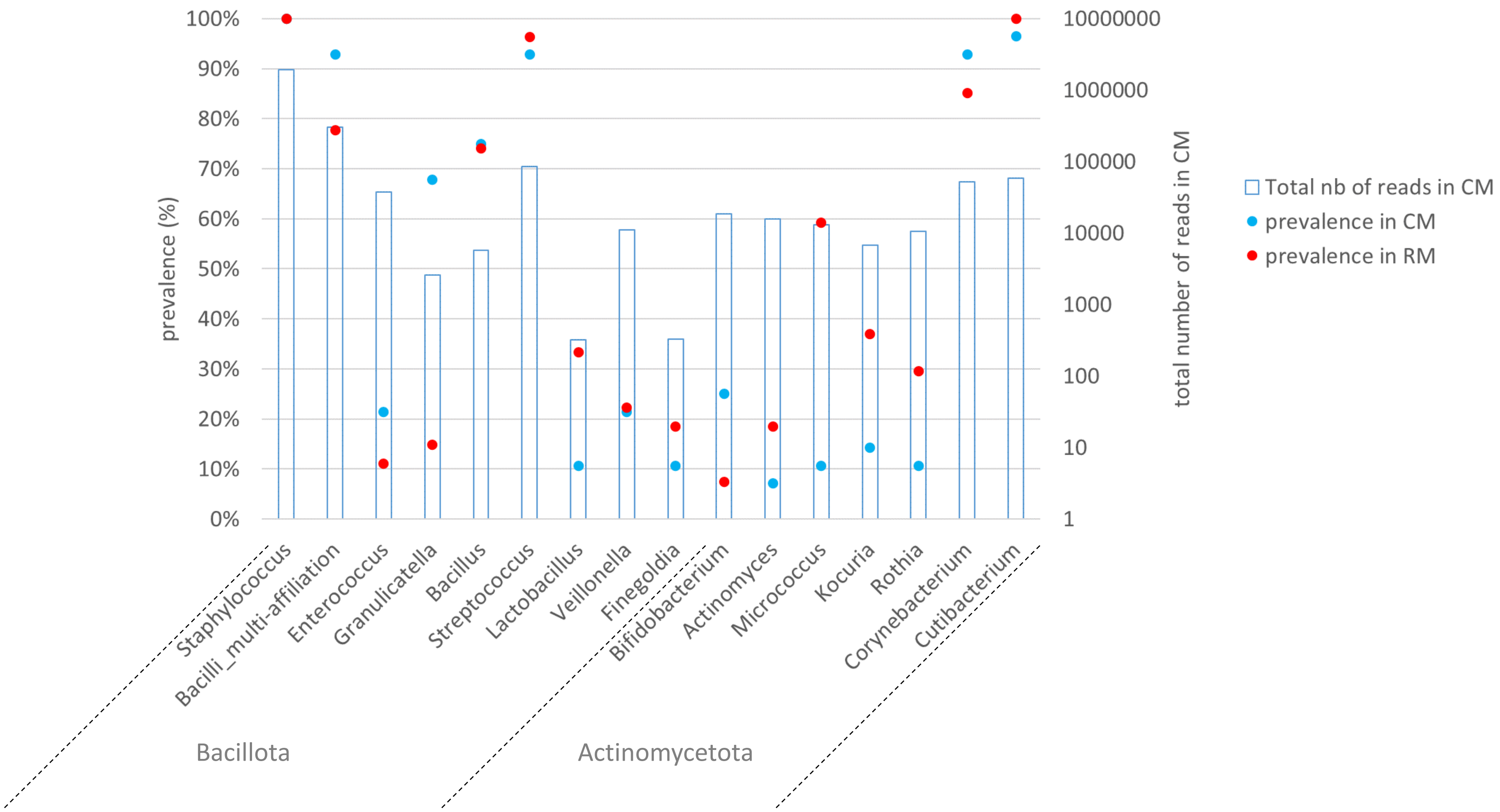

Supplementary Figure S1. Prevalent genera in the cultivable milk microbiota

Genera with prevalence in the cultivable milk microbiota (●) higher than 7% (corresponding to a minimum of 2 HM samples) and with a total relative abundance in CM higher than 0.00005 (bar chart) are presented, as well as the prevalence of these genera in the raw milk microbiota (RM) (●).
