# Supplementary material for: New insights into the cultivability of human milk bacteria from ingestion to digestion and implications for their Immunomodulatory properties
Source: Sci Rep. 2025 Mar 31;15:10985. doi: 10.1038/s41598-025-95668-6 (PMC11958788; doi:10.1038/s41598-025-95668-6)
Supplement: Supplementary file 3 — Supplementary Material 3 [file 41598_2025_95668_MOESM3_ESM.pdf]

Supplementary Table S2. Cohort characteristics

metadata	sample size
HM sample	n=28
median age (min-max)	31.5 y (21-39)
median height (min-max)	166 cm (130-177)
median weight (min-max)	63 kg (48-91)
median BMI (min-max)	23 (18-44.4)
Parity	primiparous (n= 16), multiparous (n=12)
number of lactations	One (n=16), two (n=9), three (n=3)
Week (W) of lactation milk was collected	2nd (n=3), 3rd (n=13), 4th (n=8), 5th (n=4)
Feeding mode	exclusive breastfeeding (n=25), pumped breastmilk (n=1), breasmilk provided at breast or pumped (n=2)
iron supplementation during gestation	supplementation (n=21), no supplementation (n=6), no information (n=1)
vitamin and trace elements supplementation during gestation	supplementation (n=7), no supplementation (n=21)
maternal iron supplementation during breastfeeding	supplementation (n=5), no supplementation (n=22), no information (n=1)
maternal vitamin and trace elements supplementation during breastfeeding	supplementation (n=6), no supplementation (n=22)
median population on BHIYEc under aerobic growth condition	3.19 Log cfu/ml
median population on BHIYEc under anerobic growth condition	3.44 Log cfu/ml
maternal diet	classic (n=25), vegetarian (n=3)
